# The Role of Macrophages in Cancer Development and Therapy

**DOI:** 10.3390/cancers13081946

**Published:** 2021-04-18

**Authors:** Ewa Cendrowicz, Zuzanna Sas, Edwin Bremer, Tomasz P. Rygiel

**Affiliations:** 1 Department of Hematology, University Medical Center Groningen, University of Groningen, 9713 GZ Groningen, The Netherlands; e.krol@umcg.nl (E.C.); e.bremer@umcg.nl (E.B.); 2 Department of Immunology, Medical University of Warsaw, Nielubowicza 5 Street, Building F, 02-097 Warsaw, Poland; z.sas@cellis.eu

**Keywords:** tumor-associated macrophages, immunotherapy, tumor microenvironment, tumor, immune suppression, macrophage

## Abstract

**Simple Summary:**

Tumor-Associated Macrophages (TAMs) play an important role in the development of tumors, modulation of neoangiogenesis, immune suppression, and metastasis. High infiltration of macrophages in the tumor is also correlated with poor prognosis in several cancer types. Therefore, they became an attractive target for cancer immunotherapies. In this review, we describe the role of macrophages in tumorigenesis and summarize the most recent advances in the therapies targeting TAMs.

**Abstract:**

Macrophages are critical mediators of tissue homeostasis and influence various aspects of immunity. Tumor-associated macrophages are one of the main cellular components of the tumor microenvironment. Depending on their activation status, macrophages can exert a dual influence on tumorigenesis by either antagonizing the cytotoxic activity of immune cells or, less frequently, by enhancing antitumor responses. In most situations, TAMs suppress T cell recruitment and function or regulate other aspects of tumor immunity. The importance of TAMs targeting in cancer therapy is derived from the strong association between the high infiltration of TAMs in the tumor tissue with poor patient prognosis. Several macrophage-targeting approaches in anticancer therapy are developed, including TAM depletion, inhibition of new TAM differentiation, or re-education of TAM activation for cancer cell phagocytosis. In this review, we will describe the role of TAMs in tumor development, including such aspects as protumorigenic inflammation, immune suppression, neoangiogenesis, and enhancement of tissue invasion and distant metastasis. Furthermore, we will discuss therapeutic approaches that aim to deplete TAMs or, on the contrary, re-educate TAMs for cancer cell phagocytosis and antitumor immunity.

## 1. Introduction

The recent decade has seen major advances in understanding the role of innate and adaptive immunity in cancer, which has catalyzed the development of new cancer immunotherapeutics. A frontrunner in this respect has been the therapeutic targeting of T cells, with curative treatments such as Chimeric Antigen Receptor (CAR) T cell [1] and checkpoint inhibitor therapies [2]. However, macrophages can represent more than 50% of tumor-infiltrating immune cells. More recently, they also gained prominence, with, e.g., complete responses in a recent clinical trial in relapsed/refractory patients upon inhibition of the macrophage checkpoint CD47/SIRPα [3] as well as preclinical advances in the development of CAR macrophages [4].

Macrophages are innate immune cells pivotal for tissue homeostasis, removal of superfluous cells, and inflammatory responses to infections. Macrophages also play diverse roles in cancer development, ranging from antitumor activity in early progression stages to, most commonly, tumor-promoting roles in established cancer [5]. Notably, macrophages are highly plastic cells and, depending on the microenvironmental cues in the Tumor Microenvironment (TME), can undergo marked changes in their function. In established cancers, high macrophage infiltration often strongly associates with poor prognosis or tumor progression in many types of solid tumors, including breast [6], bladder [7], head and neck [8], glioma [9], melanoma [10], and prostate cancer [11]. Conversely, in colorectal and gastric cancers, high macrophage infiltration correlates with a better prognosis [12]. These apparently opposite effects are likely related to macrophage plasticity and resultant heterogeneity of phenotype and functions in various cancers.

Such macrophage heterogeneity has historically and simplistically been defined into a dichotomous classification of a classically activated so-called M1 subtype and an alternatively activated M2 subtype. In a recent review that combined 300 studies, a clear prognostic association was presented for various solid cancer types and the infiltration of either M1 or M2 macrophage subtypes, with the M2-subtype corresponding with poor patient outcome, contrary to the presence of the M1 subtype macrophages corresponding with a favorable prognosis [13].

In the current review, we will briefly detail the basics of macrophage biology, then provide an in-depth discussion on the diverse impact of macrophages in the tumor microenvironment and finally focus on recent advances in the therapeutic targeting of macrophages for cancer therapy.

## 2. Macrophage Activation

**Figure 1:**
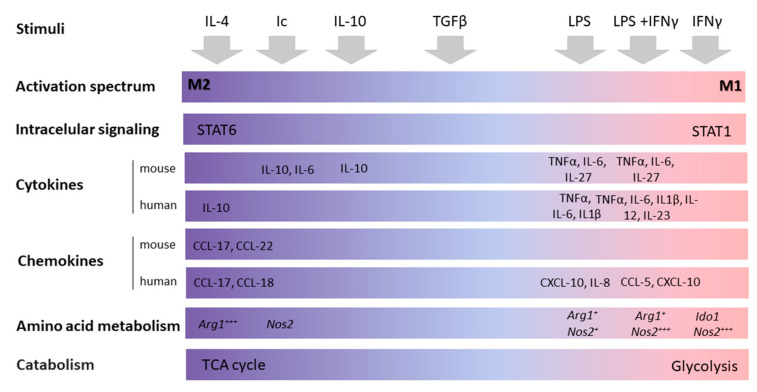
A spectrum of macrophage activation. The functional M1-M2 spectrum subdivisions of activated macrophages, derived from human monocytes or mouse bone marrow upon CSF-1 stimulation. Stimulation conditions are IL-4, immune complexes (Ic), IL-10, glucocorticoids (GC) with TGFβ, glucocorticoids alone, LPS, LPS and IFNγ, and IFNγ alone.

Similar to the Th1/Th2 T cell distinction, the diverse functions of macrophages were categorized into two opposite phenotypes, “classically activated macrophages” (M1) and “alternatively activated macrophages” (M2). M1 macrophages have a proinflammatory phenotype with pathogen-killing abilities, production of proinflammatory cytokines, like Tumor Necrosis Factor α (TNFα), Interleukin-1β (IL-1β), IL-12, IL-23 [14], secretion of reactive oxygen species (ROS) [14], and higher antigen-presenting capacities [15]. M1 macrophages can be easily generated in vitro by stimulation with Interferon-γ (IFNγ), Lipopolysaccharide (LPS), or IFNγ/LPS. Substantial overlap exists between in vivo M1 and in vitro classically activated (LPS+IFNγ) macrophages. It includes many interferon-induced genes like Irf9, Irf7, Ifi35, Ifnar2, pro-M1 genes such as Il12a, Il12b, Jak2, Stat1/Stat2, and costimulatory molecules such as CD86 and CD40 [16]. On the other hand, the M2 subtypes have higher phagocytic activity, increased expression of scavenger receptors, increased arginase pathway activity, secrete IL-10, Transforming Growth Factor β (TGFβ), and vascular endothelial growth factor (VEGF). Thus, M2 macrophages are anti-inflammatory and play a crucial role in the anti-parasitic immune response, promote tissue remodeling, neoangiogenesis, and tumor progression [17]. M2 could be generated by a range of various stimuli, including IL-4, IL-10, IL-13, or TGFβ (Figure 1). A small overlap exists between in vivo M2 (LPS-deprived) and in vitro alternatively activated macrophages (polarized with IL-4). They include the expression of genes involved in the positive regulation of the MAPK cascade (Gab1, Jun, P2ry1) and glutamine synthetase (Glul), a gene associated with glutamine metabolism [16]. However, more genes are regulated in opposite or unrelated ways. For example, in contrast to in vivo M2, the in vitro IL-4-derived alternatively activated macrophages express only the standard IL-4 markers like CD206, CD36, CD9, CD74, Bcl2, and Arg1 [16]. Macrophage activation leads to profound changes in their cellular metabolism. M1 activation is characterized by glycolysis and fatty acid synthesis, whereas M2 activation is linked with tricarboxylic acid cycle, fatty acid oxidation, and glutaminolysis (Figure 1) [18].

Despite the general acceptance, the M1/M2 dichotomic model is an oversimplification representing two phenotypic extremes of the M1-M2 spectrum, which was explained by the nomenclature and experimental guidelines published by Murray et al. [19]. However, the M1/M2 nomenclature was extensively used in multiple papers that are discussed in this review, justifying its use.

Macrophage activation state is an important aspect in tumor development and therapy. TAMs can directly inhibit cytotoxic T lymphocyte (CTL) responses through the expression of immune checkpoint molecules (e.g., programmed cell death ligand 1 (PD-L1)), production of inhibitory cytokines such as IL-10 and TGFβ [20], and metabolic activities, including the depletion of metabolites, such as L-arginine [21]. Additionally, M2 TAMs control the TME by recruitment of immunosuppressive populations such as regulatory T cells (Treg), inhibition of dendritic cells (DCs) remodeling the extracellular matrix (ECM), and the expression of various chemokines. M2 TAMs also upregulate receptors involved in the formation of ‘don’t eat me’ signaling [22]. On the other hand, M1 TAMs could take part in a robust adaptive anticancer immune response, e.g., by enhancing antigen presentation and activation of adaptive immunity. However, more frequently, particularly in the presence of tumor hypoxia, TAMs are programmed to drive immune suppression and tissue remodeling. Thus, the main therapeutic strategies to target TAMs in the TME are depletion of TAMs, reactivation into a more pro-inflammatory (M1) state, or reactivation of anticancer activity by breaking the ‘don’t eat me’ signaling.

## 3. The Role of Macrophages in Cancer Development

### 3.1. The Role of Macrophages in the Tumor-Promoting Inflammation

**Figure 2:**
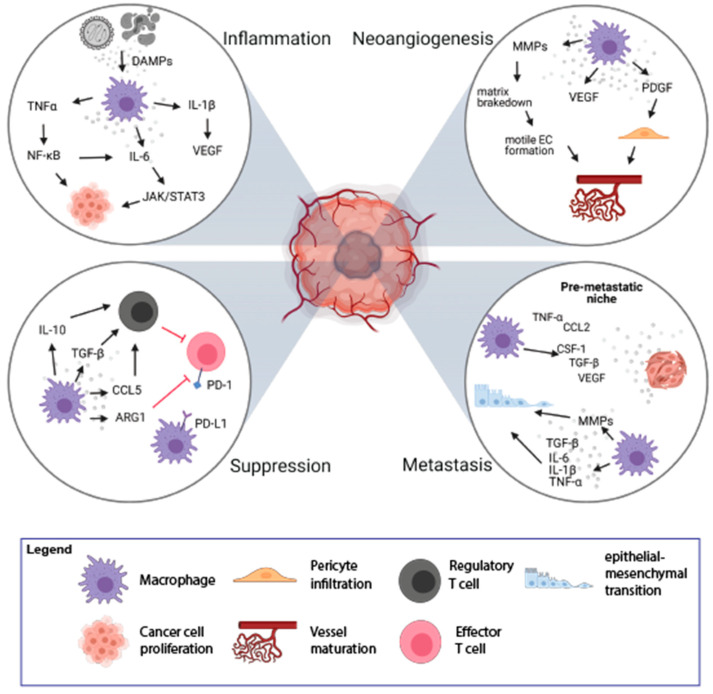
Mechanisms of tumorigenesis stimulation by TAMs. TAMs play an important role in the process of tumorigenesis by induction of inflammation (top left loop), stimulation of neoangiogenesis (top right loop), immune suppression (bottom left loop), and induction of metastasis (bottom right loop). The figure was created with Biorender.com.

In a physiological context, inflammation is initiated to restore homeostasis after the disturbance caused by external factors [23]. However, not every type of inflammation is advantageous, and chronic inflammation increases the chances for the transformation into a malignant cell. Tumor-promoting inflammation could be induced long before tumor formation and can support tumor growth by encouraging neoangiogenesis, immune suppression, and oncogenic mutations [24]. Cell death is frequent in tumors and leads to the release of damage-associated molecular patterns (DAMPs), like High Mobility Group Box 1 (HMGB1), Heat Shock Proteins (HSPs), or ATP [25,26]. This stimulation can lead to the promotion of anti-tumor immunity, e.g., by activation of dendritic cells and macrophages. However, chronic stimulation will lead to immunosuppression mediated by increased production of IL-10, which inhibits the expression of proinflammatory cytokines and induces the formation of Tregs [27] (Figure 2).

Macrophages can contribute to tumor-promoting inflammation, e.g., by secretion of proinflammatory cytokines, like IL-6, IL-1β, TNFα. On the one hand, it can induce immune response but it can also support tumor growth and survival of malignant cells. TNFα, upon binding to its receptors (TNFR1/2), activates the nuclear factor -κB (NF-κB) pathway. NF-κB further mediates cancer cell proliferation and survival by controlling the expression of target genes (e.g., VEGF, IL-6) and stimulation of neoangiogenesis [28]. The proinflammatory effect of IL-6, mediated by the JAK/STAT3 pathway, leads to cell proliferation, differentiation, and apoptosis [23,29]. Proinflammatory cytokine, IL-1β, activates endothelial cells to produce VEGF, which supports angiogenesis, contributing to tumor invasiveness and metastasis. It also drives the expression of downstream pro-tumorigenic cytokines such as IL-6, TNFα, and TGFβ [30]. TGFβ is also produced by activated macrophages and plays a dual, pro-, or anti-inflammatory role [31,32]. In the early stages of tumor development, TGFβ promotes apoptosis and inhibits the progression of the cell cycle. In the later stages, TGFβ induces epithelial-mesenchymal transition (EMT), which enhances tumor invasion and metastasis. Increased TGFβ concentrations have an inhibitory effect on anti-tumor T-cell response [23,33]. Thus, TAMs could enhance tumor formation and progression by their inflammatory activity, particularly a chronic low-grade inflammatory state.

### 3.2. Macrophages and Neoangiogenesis

The rapid proliferation of cancer cells results in the fast growth of tumor mass and increased demand for nutrients and oxygen. Essential nutrients are delivered to the tumor by a capillary network formed in the process of neoangiogenesis. The formation of new vessels is regulated by the growth factors released by cells in the TME [34]. Due to poor regulation, the structure and function of newly formed vessels are abnormal with increased vessel permeability, which contributes to disease progression [35]. Hypoxic regions of tumor tissue are formed due to the rapid and uncontrolled cell growth and are accompanied by an increased rate of cancer cell death. TAMs infiltrate these hypoxic regions to regain homeostasis through stimulation of new blood vessel formation. The process of neoangiogenesis is modulated by many factors produced by TAMs, including VEGF, matrix metalloproteinases (MMPs), platelet-derived growth factor (PDGF), and angiopoietin-1 (Figure 2) [36,37]. VEGF induces proliferation and maturation of endothelial cells by engaging the VEGF Receptor 2 (VEGFR2) expressed on the endothelial cells (ECs) [37]. VEGF also stimulates the chemotaxis of macrophages and ECs. This process is promoted by MMP-2, MMP-7, MMP-9, which are also secreted by TAMs. The main role of MMPs is to break down the extracellular matrix, which allows migration of ECs and the formation of new vascular sprouts [38]. Additionally, it facilitates the infiltration and invasion of adjacent tissues, which may also promote the formation of metastases [35].

TAMs and platelets are also the main sources of PDGF, which induces infiltration of pericytes [39]. The interaction between pericytes and ECs is crucial for vessel maturation and remodeling, which affects vascular permeability [40]. The angiopoietin-1 released from pericytes binds to Tie-2 receptor on ECs, leading to tightening of ECs’ cell-cell junctions and stabilization of newly formed vessels (Figure 2) [41].

It has been shown that a specific subset of monocytes expressing the Tie-2 receptor (Tie-2 receptor-expressing monocytes—TEMs) account for most of the proangiogenic activity of macrophages in both spontaneous and orthotopic tumors. TEMs are present in peripheral blood and are responsible for early angiogenic responses. Thus, it is thought that TEMs can be precursors of proangiogenic TAMs [42].

### 3.3. Immune Suppression and Orchestration of the Tumor Microenvironment by TAMs

The TME is infiltrated with various immune cells, out of which TAMs are the most abundant cell population. TAMs play a significant role in immunosuppression and tumor progression by releasing immunomodulatory factors such as PGE2, IL-10, and TGFβ, which inhibit cytotoxic activity of T lymphocytes and NK cells (Figure 2) [24,33]. Upon secretion of IL-10 and TGFβ, TAMs induce Tregs that suppress the activity of effector T lymphocytes. Moreover, TAMs recruit Tregs to the TME by secretion of chemokines CCL5, CCL20, and CCL22 [43]. Additionally, TAMs are involved in the conversion of Th cells into Tregs, which further inhibit the immune response in an antigen-specific manner [44,45].

The other mechanism of the suppression of the immune response can be mediated by direct cell-to-cell contact between macrophages and other immune cells. TAMs could directly inhibit the immune response by expression of surface proteins, PD-L1, CD80/CD86, or death receptor ligands, FasL or TRAIL, that function as agonists for inhibitory receptors, PD-1, CTLA-4, FAS, and TRAIL-RI/-RII, respectively, that are present on the immune effector cells [5,46]. The stimulation of PD-1 and CTLA-4 receptors leads to the inhibition of the signaling pathway from the T cell receptor (TCR) and causes a decrease in the production of cytokines and proteins that promote cell survival. PD-L1 expression has been observed on macrophages and dendritic cells in many cancer types [47] as well as on macrophages and myeloid-derived suppressor cells isolated from the hypoxic tumor regions [48]. Therefore, macrophages may modulate lymphocyte function and inhibit the antitumor immune response via PD-1/PD-L1 interaction [49]. TAMs also express CD80 and CD86, that upon binding to CTLA-4 on T lymphocytes, inhibit their activation [50]. Moreover, TAMs produce arginase-1—an enzyme degrading L-arginine, which is necessary for the expression of TCR complex, lymphocyte proliferation, development of the immunological memory [51], and T cell-mediated antitumor response [52]. L-arginine starvation leads to inhibition of T cell proliferation via G_o_–G_1_ phase blockade [53]. Thus, TAMs have pleiotropic immunosuppressive abilities that quench adaptive antitumor immunity.

### 3.4. TAMs in Tissue Invasion and Distant Metastasis

Colonization of distant organs by neoplastic cells is a multistep process. First, cancer cells acquire the ability to grow invasively; second, they penetrate the vasculature; third, they survive in the circulation; and last effectively settle in the new metastatic location [54]. TAMs are important players in almost every step of metastasis formation [54]. Activation of Toll-like receptor 4 (TLR-4) on the surface of M2-like macrophages increases the level of IL-10, which promotes the EMT program, which plays an important role in the first steps of metastases [55]. EMT can also be induced by proinflammatory cytokines (IL-6, IL-1β, TNFα) [56] and TGFβ [57] released by TAMs (Figure 2). During the EMT, epithelial cells lose cell-cell junction and acquire motile and invasive mesenchymal cell phenotype facilitating the passage through dismounted basement membranes. TAMs are also involved in the breakdown of the extracellular membrane around endothelium by the release of MMP9 and cathepsins, which results in vascular intravasation of tumor cells. Additionally, there is a positive feedback loop between macrophages and tumor cells: CSF-1 produced by tumor cells stimulates macrophage motility and secretion of EGF, which in turn supports chemotaxis of tumor cells into blood vessels [58]. TAMs support the survival of cancer cells in the circulation by the interaction of α4 integrin with vascular cell adhesion molecule-1 (VCAM-1) on the surface of cancer cells. This interaction activates the PI3K/Akt survival pathway protecting cancer cells from the pro-apoptotic activity of molecules such as TRAIL [59]. It was observed that tumor cells are in direct interaction with TAMs when crossing the endothelial cell layer into the blood vessel [60]. Interaction of macrophages with tumor cells enhances extravasation. Before metastasis is formed, local changes occur in the target tissue leading to the creation of a premetastatic niche. Increased influx of macrophages into healthy tissue is an important step preceding the formation of metastases. Macrophages are attracted to the circulation by various agents released from tumor cells, including CSF-1, CCL-2, VEGF, TNFα, or TGFβ, and accumulate at pre-metastatic sites [61]. Macrophages that appear in the site of future metastasis form migration tracks for cancer cells by remodeling of collagen fibers, which facilitates the invasion of cancer cells [62]. TAMs shape the extracellular matrix by releasing growth factors deposited in the extracellular matrix, which results in the stimulation of neoangiogenesis, extravasation, and EMT [54]. The above-mentioned processes show the role of TAMs in the enhancement of local tumor cell migration and distant metastasis formation.

### 3.5. The Role of M1 TAMs in the Elimination of Cancer Cells

Although the M2 TAMs play an important role in tumor development, M1 TAMs have been shown to effectively eliminate cancer cells. M1 polarized macrophages drive Th responses via antigen presentation more efficiently than M2 macrophages, including T cell proliferation and IFNγ secretion [63]. IFNγ-stimulated macrophages secrete IL-12 [64], which is a proinflammatory cytokine with potent antitumor activity [65] and the ability to recover costimulatory properties of TAMs for T cells [64]. M1 macrophages also secrete less VEGF, MMPs, and CCL18 than M2 macrophages [64]. What is more, TLR ligands (e.g., LPS) either alone or together with IFNγ drive M1 polarization, which further leads to the inhibition of cancer cell growth [66]. Therefore, M1 TAMs are considered tumor-suppressive, and M2 TAMs are considered tumor-promoting macrophages [14].

## 4. Targeting TAMs for Cancer Therapy

Tumors are dynamic and heterogeneous tissues that depend on the microenvironment, with a complex relationship between cancer cells and infiltrating immune cells [67,68]. Therefore, therapeutic strategies and universal protocols to treat cancer are difficult to establish and apply, and rarely single-agent treatment is effective. TAMs play a significant role in the development of resistance to many cancer therapies due to their abundance in TME and important role in tumor progression [69,70]. Therefore, targeting TAMs becomes a crucial strategy for cancer treatment. Currently, two main approaches are used to target TAMs. One is the reduction of the number of macrophages in the tumor, which involves either depletion of TAMs or inhibition of the recruitment of monocytes, which will give rise to TAMs. The second approach involves re-education of macrophages by either repolarization to proinflammatory M1 phenotype or induction of macrophage-mediated anticancer response, like phagocytosis [71]. Many drugs used to target macrophages are currently in clinical trials both as a single agent treatment and in combination therapies, with radio-, chemotherapy, or immunotherapies. A variety of formats of macrophage-targeting therapeutics are also used, starting from small chemical molecules, soluble ligands, antibodies, and nanoparticles loaded with therapeutic agents.

### 4.1. Depletion of TAMs in the TME

The first approach is to target cytokine/chemokine ligand-receptor interactions. Cytokines are considered core regulators of TME and cancer progression. They are secreted by either cancer cells or tumor-infiltrating immune cells and, upon binding to receptors expressed on immune cells, induce specific signaling, e.g., macrophage differentiation and survival in the TME. The most common chemokines that attract macrophages to the tumor are CCL2, CCL20, CCL5, and CXCL12 [72]. As the higher macrophage infiltration in the TME is often correlated with poor patient outcomes [73], depleting them by targeting cytokine/chemokine ligand-receptor interaction is an attractive strategy.

**Figure 3:**
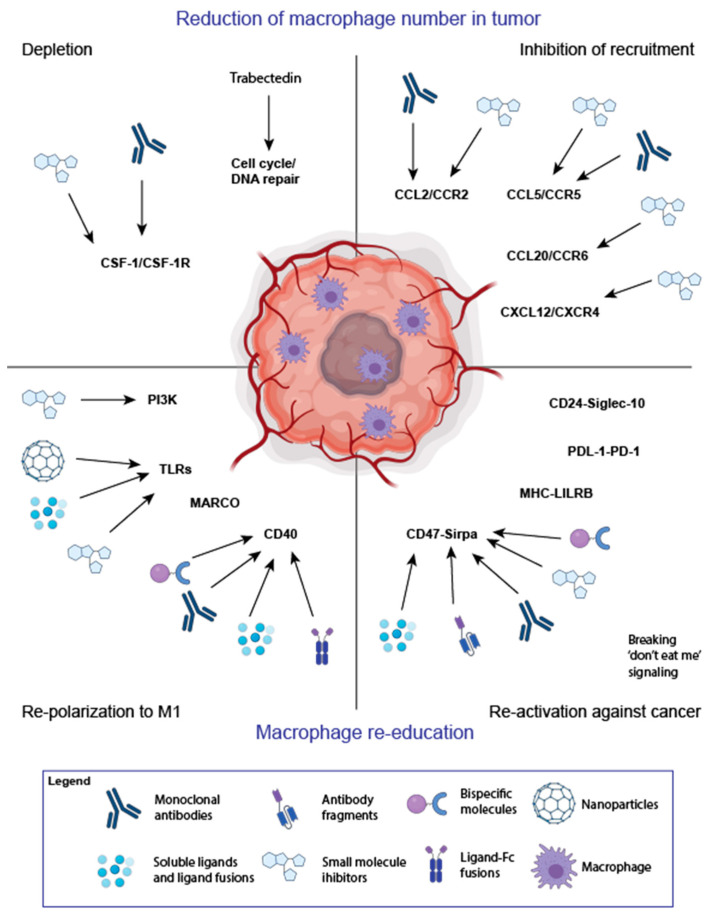
Strategies to target TAMs for anticancer therapy. Two main strategies are used to target TAMs in the TME, reduction of the number of macrophages in the tumor (top panel) or re-education of macrophages (bottom panel). Several molecular targets have been discovered to apply therapies targeting TAMs (mentioned in the figure), and various therapeutics have been developed that target these molecules, including small molecule inhibitors, monoclonal antibodies, soluble ligands, bispecific fusion proteins, and antibody fragments, and nanoparticles. The figure was created with Biorender.com.

**CSF-1R**. The main approach to deplete TAMs is to target the colony-stimulating factor 1 receptor (CSF-1R) [74]. CSF-1R belongs to a type III protein kinase receptor family and binds to two ligands, CSF-1 and IL-34. Receptor-ligand binding induces homodimerization of CSF-1R and activation of receptor signaling, which is crucial for the differentiation and survival of macrophages [74]. Most of the therapeutics that target the CSF-1/IL-34—CSF-1R pathway bind to CSF-1R. The main formats include small molecule inhibitors [75] and monoclonal antibodies (Figure 3). Many of them are summarized in a few recent reviews [74,75]. Here, we describe the most recent advances in cancer treatment with CSF-1R targeting drugs.

Pexidartinib (PLX3397) is a small molecule inhibitor targeting CSF-1R that has been approved by the Food and Drug Administration (FDA) in 2019 for the treatment of symptomatic tenosynovial giant cell tumor. Pexidartinib is studied in several clinical trials for the treatment of advanced solid tumors, recurrent glioblastoma, and hematological malignancies (NCT04703322) [76] (Table 1). Other small molecule inhibitors of CSF-1R, like ARRY-382 or DCC-3014, were well tolerated in Phase 1 trials by patients with advanced solid tumors and entered Phase 2 trials (clinical trials: NCT02880371—ARRY-382 in combination with Pembrolizumab for treatment of advanced solid tumors; NCT03069469—DCC-3014 monotherapy for the treatment of advanced tumors and tensynovial giant cell tumor). Recently, TD-92 (Erlotinib derivative) showed efficacy in the non-small lung carcinoma cancer model. The mode of action of TD-92 is different from the above-mentioned CSF-1R inhibitors. It decreases the expression of CSF-1R, which results in the reduction of the number of TAMs [77]. Monoclonal antibodies targeting CSF-1R, like AMG820, LY3022855, emactuzumab, also entered Phase 1 of clinical trials and were well tolerated by patients with a range of advanced solid tumors (Table 1). However, results from these studies vary significantly among drugs, cancer types, and combination therapies [78,79]. No sufficient anticancer activity with the use of monoclonal antibodies as a single-agent treatment has been achieved thus far for most of them [80,81].

Only a few therapeutics that are currently studied in clinical trials are targeting CSF-1, and there are no drugs in development that target IL-34. However, this molecule is getting more attention recently [82]. As CSF-1R binds to two different targets (although with different binding domains), targeting only one may not be fully effective as the other one may replace its function. In fact, in a murine model, combination treatment with antagonistic antibodies targeting both CSF-1 and IL-34 had a synergistic effect on the elimination of tissue-resident macrophages [83].

Of note, the mode of action of CSF-1R targeting compounds seems to act not only by the depletion of TAMs as previously thought. In recent studies, CSF-1R blocking antibodies resulted in repolarization of M2 macrophages to M1 phenotype, as shown in mouse models of glioma and pancreatic cancer [84,85]. In another study, treatment with PLX3397 depleted only M2 macrophages, but CD206^+^ macrophages persisted. It also resulted in the less protumor phenotype of macrophages [85]. Therefore, CSF-1R antagonists can have a dual mode of action: Depletion of protumoral M2 macrophages and repolarization of M2 macrophages into proinflammatory M1 subtype.

**Trabectedin** is a marine-derived alkaloid that possesses a few functions: It binds a minor groove of DNA and blocks the cell cycle but also affects gene transcription and DNA-repair pathways. It was shown that it also selectively reduces the number of TAMs but does not affect neutrophils or lymphocytes in the TME. Additionally, it inhibits the local differentiation of monocytes into fully mature macrophages [86]. Treatment with trabectedin depleted monocytes and macrophages in several animal tumor models and resulted in reduced tumor growth, downregulation of neoangiogenesis, and production of IL-6, CCL2, and CXCL8 [87,88]. Trabectedin is approved by FDA for the treatment of unresectable or metastatic liposarcoma and leiomyosarcoma (Table 1) [89]. Recent clinical trials showed efficacy in the treatment of soft tissue sarcoma with trabectedin and radiotherapy and for the treatment of platinum-sensitive ovarian cancer with trabectedin and pegylated liposomal doxorubicin [90,91].

**Table 1:** List of therapeutics that target TAMs and are currently studied in clinical trials or have recently been approved for the treatment of solid tumors.

Target	Compounds	Form	(Pre)Clinical Phase	Cancer Type	Reference
**TAMs depletion/inhibition of recruitment**					
CSF-1R	Pexidartinib/ PLX3397	Small molecule inhibitor	Approved by FDA in 2019 for the treatment of symptomatic tenosynovial giant cell tumor (TGCT)	Tensynovial giant cell tumor Studied in clinical trials in advanced solid tumors	[92]
	ARRY-382	Small molecule inhibitor	Phase 1b/2		[93]
	DCC-3014	Small molecule inhibitor	Phase 1/2	Advanced solid tumors	[94]
	AMG820 mAb	Monoclonal antibody	Phase 1/2 as monotherapy and in combination with pembrolizumab	Advanced solid tumors	[80,81]
Cell cycle/DNA repair	Trabectedin	Small molecule	Approved for the treatment of liposarcoma and leiomyosarcoma	Liposarcoma, leiomyosarcoma Studied in the treatment of ovarian cancer (combination therapy)	[89,95,96]
CCL2/CCR2	propagermanium	Small molecule inhibitor	Phase 1	Breast cancer	[97]
	CNTO888	Anti-CCL2 Monoclonal antibody	Phase 1	Solid tumors	[98]
CCL5/CCR5	Maraviroc	Small molecule inhibitor	Phase 1	Metastatic colorectal cancer	[99]
	Vicriviroc	Small molecule inhibitor	Phase 2	Advanced metastatic colorectal cancer	[100]
	Leronlimab	Anti-CCR5 monoclonal antibody	Phase1	Triple-negative breast cancer	[101]
**TAMs reprogramming/re-activation**					
CD40	ChiLob7/4	Chimeric monoclonal antibody	Phase 2	pancreatic cancer and head and neck cancer	[102]
	CDX-1140	Antibody	Phase 1	Melanoma, advanced cancers	[103]
	Sotigalimab (APX005M)	Humanized rabbit IgG1 monoclonal antibody	Approved with orphan drug status	Orphan drug status for the treatment of gastroesophageal junction cancer and pancreatic cancer.	[104]
	ABBV-428	Mesothelin-CD40 bispecific	Phase 1	Advanced solid tumors	[105]
TLRs	Resiquimod	Small molecule targeting TLR 7/8	Phase 1/2	melanoma	[106]
PI3K	IPI-549	Small molecule inhibitor	Phase 1b	Advanced solid tumors	[107]
CD47/ SIRPα	Magrolimab	Monoclonal antibody	Phase 1–3	Solid tumors and hematological malignancies	[108]
	TTI-621	SIRPα-Fc	Phase 1	Hematological malignancies	[109]

### 4.2. Targeting Macrophage-Recruiting Chemokines

**CCL2/CCR2**. CCL2 is a chemokine that attracts high CCR2-expressing monocytes to the tumor site. Increased expression of CCL2 positively correlates with the accumulation of macrophages in many solid tumor types [72,110,111]. The increased level of CCL2 is associated with metastasis of many cancers [112] and is a negative prognostic factor for several cancer types [113,114]. The role of CCR2 seems to outreach the influence on TAM recruitment at the tumor site. In a recent study, it has been proven that CCR2 is involved in the recruitment and initiation of tumor-promoting inflammation [115]. To break the CCL2-CCR2 interaction and inhibit monocyte recruitment to the TME, both CCR2 and CCL2 antagonists are used (Figure 3). Many preclinical studies showed high efficacy of CCL2/CCR2 antagonists, e.g., in the mouse model of lung adenocarcinoma, targeting CCR2 with a small molecule inhibitor not only reduced recruitment of M2-type macrophages but also induced tumor infiltration of activated CD8^+^ T cells [116]. Administration of CCL2 neutralizing antibodies reduced tumor growth, inhibited angiogenesis, and macrophage infiltration in a mouse model of clear cell renal cell carcinoma [117]. Many other preclinical studies on hepatocellular carcinoma [114], prostate cancer [118], and breast cancer showed that either depletion of CCR2 or breaking the CCL2-CCR2 interaction has an impact on inhibition of TAMs recruitment and tumor regression or inhibition of metastasis [119].

Several monoclonal antibodies and small-molecule inhibitors targeting CCL2/CCR2 pathway entered Phase 1 clinical trials: CCL2-neutralizing antibody CNTO888 (Carlumab), anti-CCR2 antibody (MLN1202, plozalizumab), and CCR2 antagonist (CCX872-B) (Figure 3 and Table 1). Even though well-tolerated, some of these therapeutics have not yet provided sufficient therapeutic effect [98,120]. Only CCX872 prolonged OS of patients with metastatic pancreatic cancer [121] in clinical trials in combination with Folfirinox. Recently, a CCR2 small molecule antagonist, propagermanium (PG), an approved therapeutic agent for hepatitis B, was used in a clinical trial in oral and gastric cancer patients. PG induced apoptosis of cancer cells and prolonged OS of refractory oral and gastric cancer patients (Table 1). However, PG also has immunomodulatory functions. Therefore, it is not clear whether it acted only via the CCR2 pathway [122].

Additionally, it was shown that other pathways are involved in the mediation of CCL2 secretion and TAM recruitment, e.g., upregulation of CtBP1 promoted activation of CCL2 secretion and, as a result, infiltration of TAMs in non-small cell lung cancer (NSCLC) [123]. Therefore, targeting other pathways may have a secondary effect on the inhibition of the influx of macrophages in the tumor.

**CCL5/CCR5** signaling plays an important role in the inflammatory response by directing immune cells to the site of inflammation. In the TME, high levels of CCL5 result in the accumulation of macrophages and lymphocytes with high CCR5 expression. Besides its role in the recruitment of immune cells, it is also involved in the process of tumor growth, induction of drug resistance, cancer stem cell expansion, cancer cell invasion, neoangiogenesis, and immunosuppressive polarization of macrophages (reviewed in [124]). In the preclinical studies in pancreatic and prostate cancer mouse models [125,126], CCR5 antagonists reduced tumor growth, adhesion, and invasion. CCR5 is targeted by several antagonists: Humanized monoclonal antibodies like PRO 140 (Leronlimab), small molecule inhibitors like Maraviroc, Vicriviroc, BMS-813160, or TAK-779 [127]. All these drugs were developed for the treatment of HIV infections [128]. However, they are potential candidates to target TAMs in cancer therapy. Recently, Maraviroc was used in Phase 1 clinical trial in combination with Pevrolizumab in the treatment of metastatic colorectal cancer with a good toxicity profile [99] (Table 1) and is currently used in a clinical trial with nivolumab and ipilimumab for the treatment of metastatic colorectal and pancreatic cancers (clinical trial: NCT04721301). Leronlimab is currently studied in Phase 1 in combination with carboplatin in the treatment of triple-negative breast cancer [101] and in Phase 2 for the treatment of solid metastatic tumors (NCT0450494). The safety and efficacy of Vicriviroc in combination with pembrolizumab is studied in Phase 2 in patients with advanced metastatic colorectal cancer (reviewed in [124]). Other approaches to target the CCL5/CCR5 pathway are also developed, e.g., short-interfering RNA (siRNA) [129] or zinc finger nuclease [130]. In one study, a saponin, DT-13 reduced CCR5 expression and gastric cancer cell migration in a preclinical model [131].

CCL20, also known as macrophage inflammatory protein-3α (MIP-3α), is a chemokine ligand for the CCR6, expressed on dendritic cells, regulatory T cells, T helper lymphocytes, neutrophils, and macrophages that stimulates their migration and function [132]. It was shown that CCR6 was upregulated on TAMs and promoted the recruitment of proinflammatory macrophages in the mammary tumor microenvironment. TAMs recruitment via CCR6 facilitated the onset of neoplasia in vivo [133]. Moreover, high CCL20/ CCR6 levels relate to stage and prognosis in many cancer types, including breast cancer [134], glioma [135], colorectal cancer [136], and non-small lung cancer [137]. Several monoclonal antibodies, small molecule inhibitors, and CCR6 targeting peptides are being developed. Although most of them are developed to treat various types of inflammatory diseases, e.g., CCX9664, a small molecule antagonist of CCR6, is being studied in rheumatoid arthritis [138], they are also potential candidates for the treatment of cancer [138] (Figure 3).

**CXCL12/CXCR4.** CXCL12 is another chemokine, which regulates the migration of monocytes [139]. Elevated CXCR4 is correlated with the tumorigenesis of NSCLC [140]. It was shown that CXCL12 secretion could be induced in response to radiation therapy and cause accumulation of TAMs in the tumor [72]. Of note, CXCL12 works synergistically with CCL2 to enhance the migration of human monocytes and macrophages [141]. One of the CXCR4 antagonists is Plerixafor (AMD3100). It was approved in 2008 for mobilization of hematopoietic stem cells for autologous transplantation, and it is being studied in clinical trials in various cancer types. For example, it is used in combination with chemo-radiotherapy for the treatment of glioblastoma and studied for its ability to prevent recurrence of glioblastoma after radiation treatment (clinical trial: NCT03746080). Another CXCR4 antagonist, BL-8040 (motixafortide), showed efficacy in combination with pembrolizumab and chemotherapy in pancreatic ductal adenocarcinoma [142].

### 4.3. Repolarization and Re-Education of TAMs against Cancer Cells

Many therapeutic strategies focus on the depletion of TAMs. However, many of these approaches were not fully successful in clinical studies, especially as single-agent therapy [80,81]. TAM depletion may be effective as a combination therapy with chemo, radio, or immunotherapy. Moreover, recent findings suggest that re-education of TAMs rather than depletion may represent a more effective strategy.

**CD40** is a member of the tumor necrosis factor receptor (TNFR) superfamily and is broadly expressed on APCs, including monocytes, macrophages, and dendritic cells, and is upregulated on macrophages upon activation. Upon interaction with its ligands (CD40L or CD154), CD40 induces the production of IL-12 and costimulatory molecules B7-1 and B7-2, which are necessary for the activation of effector CD8^+^ T cells [143]. Therefore, the most attention for CD40 agonists was focused on the induction of adaptive immune responses. However, upon CD40 costimulation, macrophages secrete NO, IL-12, and IFNγ, which are characteristic for proinflammatory M1-like activation state that leads to apoptotic-destruction of cancer cells in vitro [144] and in vivo independent on T cell activity [144,145].

Several formats of therapeutics targeting CD40 have been designed, including recombinant human CD40 Ligand (CD40L) and its fusions, CD40L gene therapy using adenoviral vectors expressing CD40L, and agonistic CD40 antibodies (Figure 3). However, some soluble ligands are not fully effective because the costimulation of CD40 requires cross-linking. Therefore, the second generation of CD40-targeting drugs is being developed, containing an Fc-domain to provide cross-linking via FcγR and improve efficacy. Tumor-targeted bispecific molecules like ABBV-428, a mesothelin-CD40 bispecific are also developed and may provide cancer-targeted activation of macrophages. ABBV-428 is currently being studied in Phase 1 clinical trial as monotherapy or in combination with nivolumab for the treatment of patients with advanced solid tumors (clinical trial: NCT02955251). Recently, CAR T cells secreting CD40 antibodies showed efficacy in a human ovarian cancer xenograft model in vivo [146]. Currently, at least ten therapeutics targeting CD40 are being studied in clinical trials. Some of them entered Phase 2, including ChiLob7/4 for the treatment of pancreatic cancer and head and neck cancer (NCT01561911), APX005M for the treatment of non-small cell lung cancer (NCT03123783), soft tissue sarcoma (NCT03719430), or advanced melanoma or renal cell carcinoma (NCT04495257), and CDX-1140 [147] for the treatment of melanoma and advanced tumors (NCT03329950, NCT04364230) [103] (Table 1). Most of the drugs are studied as monotherapy, in combination with PD-L1/PD-1 targeting antibodies or chemotherapy. APX005M has recently been approved by FDA for the treatment of esophageal and gastroesophageal junction cancer and pancreatic cancer with an orphan drug status.

**Toll-like receptors (TLRs)** sense conserved molecular patterns, like pathogen-associated molecular patterns (PAMPs) or DAMPs. Their activation could initiate an immune response. TLRs play many roles in the activation of macrophages: They regulate cytokine production, survival, play a role in recognition of self and non-self antigens, and invading pathogens [148]. Many therapeutics are designed to target TLRs to repolarize macrophages from M2-like to M1-like activation state [149] and boost the immune response against cancer cells. One example of such an immunostimulatory drug targeting TLR7/8 is resiquimod (R848), which showed an immunomodulatory effect in patients with melanoma and cutaneous T cell lymphoma with topical application of the drug [106,150] (Table 1). Additionally, soluble R848 or in nanoparticle-formulation was effective in several preclinical tumor models, resulting in improvement of survival in the murine pancreatic model [151], improvement of the efficacy of chemotherapy [152], repolarization of M2 into M1, and enhancement of antibody-dependent cellular phagocytosis [153], and reshaping of the myeloid compartment in TME leading to tumor regression [154]. Targeting other TLRs also results in the stimulation of macrophages and dendritic cells. For example, Pam3CysSK4 peptide, a ligand for TLR-2, activated dendritic cells and primed CD8^+^ T cells in a mouse model [155] and improved the efficacy of CTLA-4 immunotherapy in the melanoma mouse model [156]. TLR-3 was targeted with TLR-3 ligand (TLR-3L) and resulted in reprogramming of M2 macrophages towards M1 activation and inhibition of tumor growth [157]. Treatment with TLR-4 agonist, E6020, increased efficacy of trastuzumab and protected mice after re-challenge with HER2-positive cancer cells [158]. Recently, it was shown that Paclitaxel (a cytostatic drug used in cancer treatment) also acts via TLR-4 to reprogram TAMs toward M1 phenotype [159]. Many therapeutics are being in the developmental stage, and TLR-targeted therapy can be effective alone or can boost immunotherapy with monoclonal antibodies and immune checkpoint inhibitors (Figure 3).

**MARCO**. Another target molecule is the Macrophage Receptor with Collagenous Structure (MARCO). MARCO is expressed on M2 macrophages in the TME with an immunosuppressive gene profile [160]. The presence of MARCO-expressing TAMs is correlated with an increased number of regulatory T cells and anti-inflammatory cytokine IL-37 and diminished activity of CD8^+^ T cells, and decreased the number of NK cells [161]. Targeting MARCO with monoclonal antibody reduced tumor growth and impaired metastasis in a murine model of melanoma, colon, and breast cancer [162].

**Zoledronic acid (ZA)** is an approved medication used to treat various bone diseases and to prevent skeletal fractures in patients with some types of cancer. In vitro analysis revealed that ZA decreased the expression of M2 macrophage markers CD206, TGFβ, and Arg-1 [163] and inhibited differentiation of monocytes to macrophages in mesothelioma [164]. However, the exact mechanism of action of ZA on macrophages is not well understood.

**PI3K**.Using human and syngeneic animal models, Kaneda et al. showed that macrophage PI3Kγ/Akt signaling inhibits NFκB activation and promotes immune suppression during inflammation and tumor growth. Conversely, inhibition of macrophage PI3Kγ stimulates NFκB activation and promotes an immunostimulatory transcriptional program that restores cytotoxic T cell activity and induces tumor growth inhibition [165]. Selective PI3Kγ inhibition using drug IPI-549 is now under investigation in several clinical trials for anticancer therapy (NCT03961698, NCT03980041, NCT03795610).

Another promising approach is to induce an anticancer immune response by a genetic modification of macrophages. This approach was used by Brempelis et al., where macrophages were transduced using lentivirus to express IL-12 [166]. Adoptively transferred engineered macrophages infiltrated experimental tumors and decreased their size, activating T cells and IFNγ production [166]. An alternative approach was executed by Zhang et al. using transient genetic modification of TAMs. In that study, lipid nanocarriers were used in vivo to deliver stabilized mRNA to express IRF5 and IKKβ. Specific delivery was achieved by coating nanocarriers with mannose, a ligand for CD206 that is an M2 surface marker. This treatment resulted in the induction of anti-tumor immunity and promoted tumor regression in models of ovarian cancer, melanoma, and glioblastoma [167].

### 4.4. Induction of Phagocytosis

The main approach to re-activate TAMs, which will result in the direct destruction of cancer cells, is the induction of phagocytosis. Cancer cells express both pro- and anti-phagocytic signals that can either induce or inhibit phagocytosis. Often, cancer cells overexpress both types of signals. The balance between pro and anti-phagocytic signals in the tumor microenvironment results in the level of cancer cells engulfed by macrophages. Pro-phagocytic signals can originate from cancer cells and are “eat-me” ligands like SLAMF, calreticulin, or Phosphatidylserine (PtdSer), or external factors like opsonizing antibodies. “Don’t eat me signals” are proteins that are upregulated on cancer cells and help them avoid the immune response, among which the best studied are CD47, PD-L1 but also CD24 and MHC-I [168].

**CD47-SIRPα** interaction is the first discovered ‘don’t eat me’ signal in cancer. CD47 is upregulated in several solid tumor types [169,170], and hematological malignancies [171,172] and such overexpression is correlated with poor patient survival or poor response to the therapy. The binding of CD47 expressed on cancer cells to SIRPα expressed on macrophages inhibits phagosome formation preventing the engulfment of cancer cells. CD47 antagonists enhance not only phagocytic uptake of cancer cells by macrophages but also antigen presentation, which further triggers cross-priming of T cells [173]. At least 10 therapeutics that block CD47:SIRPα interaction have been developed and are being studied in clinical trials, including magrolimab with completed Phase 1/2 in solid tumors and hematological malignancies and has entered Phase 3 in combination with Azacitidine for treatment of Myelodysplastic Syndrome (MDS) (clinical trials nr NCT04313881) [3,108] (Table 1). Another promising therapeutic is SIRPα-Fc (TTI-621) with completed Phase 1 in B cell non-Hodgkin Lymphoma [109]. Other drugs are summarized in a recent review with a detailed description of outcomes from clinical trials [3]. Since the first successful outcomes reached by magrolimab, many therapeutics targeting CD47-SIRPα have been developed, including monoclonal antibodies, SIRPα fusions [174], bispecific antibodies and molecules, and small molecule inhibitors, which directly block the interaction between CD47 and SIRPα (Figure 3). An interesting example is RRx-001, which downregulates CD47 on cancer cells and SIRPα on monocytes and stimulates TAMs against cancer cells. It has reached Phase 3 clinical trial in small cell lung cancer [175,176,177,178] (NCT03699956). RRx-001 is also studied in clinical trials for the treatment of patients with small cell carcinoma, neuroendocrine tumors, or ovarian epithelial cancer who have failed a platinum based doublet regimen (clinical trial: NCT02489903).

**CD24-Siglec-10**. CD24 is a mucin-like GPI-anchored molecule with a very broad role in the development of cancer. CD24 is often overexpressed in cancer, and its overexpression is correlated with poor prognosis in various cancer types [179]. Moreover, CD24 regulates cell proliferation, migration, and invasion, e.g., CD24^+^ cells promote invasion and metastasis in osteosarcoma [180]. It is also a putative marker for cancer stem cells [181]. Moreover, its role in the inhibition of macrophages was recently discovered. It was shown that upon interaction with Siglec-10, expressed on TAMs, CD24 serves as an immune checkpoint and inhibits phagocytosis [22]. Therefore, it serves as a new ‘don’t eat me’ signal in cancer. Several antibodies [182], and antibody fusions (for example, with CD30 or CD20 [183]) that target CD24 have been developed. However, most of them aim to target CD24 on cancer cells and were not studied as immune checkpoint inhibitors to reactivate phagocytosis by TAMs.

Another approach to induce phagocytosis of cancer cells is the use of macrophages engineered to express chimeric TCR that is directed to a cancer-specific antigen. This approach was used by Carisma Therapeutics to produce CAR macrophages (CAR-Ms) that showed antigen-specific phagocytosis and tumor clearance in vitro. CAR-Ms demonstrated expression of proinflammatory cytokines and chemokines, recruited, and presented antigen to T cells. Notably, a single infusion of human CAR-Ms decreased tumor burden and prolonged overall survival in two tumor xenograft mouse models [4].

## 5. Future Perspectives

Targeting macrophages to treat cancer is a young but rapidly developing area of research and therapy. Despite great interest, the optimum therapeutic approach has yet to be identified. The reason for that is that TAMs represent the heterogeneous population, and their role in the tumor varies depending on many environmental conditions. The other difficulty arises from TME that is a very dynamic tissue and contains various infiltrating immune cells and external factors that influence tumor progression, macrophage polarization, and response to therapies. Some of the macrophage-targeting therapeutics were effective as monotherapy. However, more evidence exists that targeting TAMs could improve the efficacy of conventional therapies and immunotherapeutics. Currently, two main approaches that target TAMs with apparent opposite effects are developed. One is to deplete macrophages; another is to re-educate them to kill cancer. Depending on the macrophage infiltration status and chosen therapy as a combination treatment, various approaches will be chosen. For example, through their Fcγ receptors, macrophages were shown to uptake therapeutic antibodies like anti-PD-L1, limiting the efficacy of such therapeutic modalities in animal models. In fact, in several recent studies, it was shown that depleting macrophages with the use of CCL2/CCR2 antagonists improves the efficacy of PD-L1 targeting antibodies and possibly other immune checkpoint inhibitors [184,185].

Interaction of antibodies with Fc receptors from macrophages must be taken into consideration in the development of antibody-driven therapeutic modalities, particularly when these will be combined with approaches that deplete or inhibit macrophage infiltration (e.g., targeting CCR2 or CSF1R). The use of CAR-M opened a completely new avenue of adoptive macrophage therapies that may bring new developments in cancer treatment.

In addition, if TAMs antagonists are being used to overcome resistance to immunotherapy, then more clinical data that correlate macrophage infiltration and/or their phenotype with patient and therapeutic outcomes must be developed to guide patient selection and improve the use of macrophage-targeting combination therapies. Despite these difficulties, there is still a great potential in harnessing macrophages biology to improve therapies in oncology.

## Data Availability

Not applicable.
